# Multiple endocrine neoplasia type 1

**DOI:** 10.1186/1750-1172-1-38

**Published:** 2006-10-02

**Authors:** Francesca Marini, Alberto Falchetti, Francesca Del Monte, Silvia Carbonell Sala, Alessia Gozzini, Ettore Luzi, Maria Luisa Brandi

**Affiliations:** 1 Regional Center for Hereditary Endocrine Tumours, Department of Internal Medicine, University of Florence, Florence, Italy; 2 DeGene Spin-off, Department of Internal Medicine, University of Florence, Florence, Italy; 3 Department of Internal Medicine, University of Florence, Viale Pieraccini, 6, 50139 Florence, Italy

## Abstract

Multiple Endocrine Neoplasia type 1 (MEN1) is a rare autosomal dominant hereditary cancer syndrome presented mostly by tumours of the parathyroids, endocrine pancreas and anterior pituitary, and characterised by a very high penetrance and an equal sex distribution. It occurs in approximately one in 30,000 individuals. Two different forms, sporadic and familial, have been described. The sporadic form presents with two of the three principal MEN1-related endocrine tumours (parathyroid adenomas, entero-pancreatic tumours and pituitary tumours) within a single patient, while the familial form consists of a MEN1 case with at least one first degree relative showing one of the endocrine characterising tumours. Other endocrine and non-endocrine lesions, such as adrenal cortical tumours, carcinoids of the bronchi, gastrointestinal tract and thymus, lipomas, angiofibromas, collagenomas have been described. The responsible gene, *MEN1*, maps on chromosome 11q13 and encodes a 610 aminoacid nuclear protein, menin, with no sequence homology to other known human proteins. MEN1 syndrome is caused by inactivating mutations of the *MEN1 *tumour suppressor gene. This gene is probably involved in the regulation of several cell functions such as DNA replication and repair and transcriptional machinery. The combination of clinical and genetic investigations, together with the improving of molecular genetics knowledge of the syndrome, helps in the clinical management of patients. Treatment consists of surgery and/or drug therapy, often in association with radiotherapy or chemotherapy. Currently, DNA testing allows the early identification of germline mutations in asymptomatic gene carriers, to whom routine surveillance (regular biochemical and/or radiological screenings to detect the development of MEN1-associated tumours and lesions) is recommended.

## Definition

Multiple Endocrine Neoplasia Type 1 (MEN1, OMIM 131100) is a rare inherited autosomal dominant cancer syndrome with a very high penetrance and an equal sex distribution that is characterised by the presence of hyperplasia and neoplasia in at least two different endocrine tissues (parathyroid adenomas, entero-pancreatic tumours and pituitary tumours) within a single patient. Two different forms, sporadic and familial, have been described. The sporadic form presents with two of the three principal MEN1-related endocrine tumours, while the familial form (more frequent and with an autosomal pattern of inheritance) consists of a MEN1 case with at least one first degree relative showing one of the endocrine characterising tumours.

## Epidemiology

MEN1 is a rare disease that occurs in approximately one in 30,000 individuals with an equal sex distribution. The MEN1 syndrome has been described in diverse geographic regions and ethnic groups, and no racial predilection has been demonstrated.

Endocrine and non-endocrine manifestations of the disease in MEN1 patients most often begin in the fourth or fifth decade. The onset of the disease is rare before age 10 years.

## Clinical description, diagnostic methods, treatments

MEN1 syndrome is characterised by the occurrence of primary tumours involving two or more endocrine tissues within a single patient. It encompasses tumours of the parathyroids (95% of cases), pancreatic islets (from 30 to 80% of cases) and anterior pituitary (from 15 to 90% of cases). Other endocrine and non-endocrine lesions, such as adrenal cortical tumours [1,2], carcinoids of the bronchi [3], gastrointestinal tract [4] and thymus [5], lipomas, angiofibromas and collagenomas [6,7] have been described, but with a lower frequency. Combinations of over 20 different endocrine and non-endocrine tumours and lesions have been reported [8-12]. Thus, no simple definition of MEN1 could cover all index cases or all families. By definition, MEN1 should be suspected in patients with an endocrinopathy of two of the three principally affected organs, or with an endocrinopathy of one of these organs plus a first-degree relative with MEN1.

MEN1 affects all age groups with an age range of 8–81 years, and more than 95% of patients develop clinical manifestations by the fifth decade [13-15]. Hyperparathyroidism is the most common and usually the first clinical manifestation of MEN1. Gastrinoma and carcinoids represent the most frequent causes of mortality. The onset of the MEN1-associated primary hyperparathyroidism and the onset of MEN1-associated gastrinoma and insulinoma anticipate the onset of the corresponding sporadic counterparts of three and one decades, respectively.

### Parathyroids tumours

Primary hyperparathyroidism (PHPT) is the most common clinical manifestation of MEN1, affecting more than 95% of all MEN1 patients [14]. The age of the onset (typically between 20 and 25 years of age) of MEN1-associated parathyroid tumours is about three decades earlier than that of sporadic parathyroid adenoma; these tumours are generally characterised by multiglandular hyperplasia [16].

Symptoms of PHPT in MEN1 are the same as those of sporadic PHPT. PHPT in MEN1 manifests with hypercalcaemia as a result of overproduction of parathyroid hormone (PTH) by tumoural and supernumerary parathyroid glands. PHPT is defined as an increased serum concentration of PTH (normal range 10–60 pg/ml) [17] and an increased serum concentration of calcium (normal range 8.5–10.5 mg/dl or 2.1–2.6 mmol/l) [17]. The common clinical manifestations of hypercalcaemia include:

1) central nervous system – altered mental status, including lethargy, depression, decreased alertness and confusion;

2) gastrointestinal tract – anorexia, constipation, nausea and vomiting:

3) kidneys – polyuria, nycturia, polydipsia, impaired concentrating ability, dehydration, hypercalciuria and increased risk for kidney stones;

4) skeleton – increased bone resorption and increased fracture risk, mainly in women who manifest PHPT before 35 years of age;

5) cardiovascular system – hypertension, shortened QT interval.

Moreover, hypercalcaemia may increase the secretion of gastrin from a gastrinoma.

At present, total parathyroidectomy is the proposed effective treatment for PHPT in symptomatic hypercalcaemic MEN1 patients. Subtotal parathyroidectomy results in a 50% risk of recurrence within 8–12 years after the intervention. Total parathyroidectomy is often followed by autotransplantation of resected fresh or cryopreserved normal parathyroid tissue in the forearm. To prevent late recurrence, a total parathyroidectomy followed by a life-long treatment with vitamin D analogues is used as an alternative. Intraoperative rapid parathormone monitoring aids both detection of extra glands and immediate assessment of the postresection parathormone level.

### Pancreatic tumours

Pancreatic tumours occur in about 30–80% of MEN1 patients and are the second most frequently expressed clinical manifestation of MEN1. They are characterised by multiple nodular lesions developed at an early age [18,19]. The majority of these tumours produce excessive amounts of hormone (gastrin, insulin, glucagons, somatostatin, neurotensin or vasoactive intestinal polypeptide (VIP)) and are associated with distinct clinical syndromes. These hormone-secreting tumours can be detected by biochemical screening for elevated serum hormone concentrations. The most common functional pancreatic tumours are gastrinomas and insulinomas. Nevertheless, about one third of pancreatic tumours are non-functional and clinically silent. Non-functional tumours and insulinomas are located within the pancreas, while gastrinomas are often found in the soft tissue around the pancreas and in the duodenal submucosa, but not in the mucosa where the gastrin-producing G cells are located. Endoscopic ultrasonography (EUS) examination is the most sensitive imaging procedure for the detection of small (≤10 mm) pancreatic endocrine tumours in asymptomatic MEN1 patients; its sensitivity is higher than 75%. The use of EUS in association with Octreoscan scintigraphy increases the pancreatic tumoural detection rate to 90% [20]; EUS allows precise localisation of the tumours, while Octreoscan scintigraphy gives much more information about the spread of the disease and detects liver metastases with a sensitivity of 92% [21].

#### Gastrinomas

These gastrin-secreting tumours represent more than 50% of all pancreatic tumours in MEN1. Approximately 40% of MEN1 patients have gastrinoma that manifests as Zollinger-Ellison syndrome (ZES) which usually occurs before age 40 years (about one decade earlier than sporadic gastrinomas) [16]. ZES may lead to upper abdominal pain, diarrhoea, oesophageal reflux, vomiting and acid-peptic or duodenal ulcers and, more rarely, heartburn and weight loss. Gastrinomas represent the major cause of morbidity and mortality in MEN1 patients, principally due to severe multiple peptic ulcers that may perforate. Biochemical diagnosis is made by demonstration of an increased basal gastric acid secretion [22]; gastrinoma is defined as elevated basal serum concentration of gastrin (normal range <100 ng/l) [17].

The treatment for a non-metastatic gastrinoma is surgical resection. The treatment for multiple and disseminate gastrinomas consists of therapy with a human somatostatin analogue (octreotide), administration of proton pump inhibitors or H_2_-receptor blockers to reduce gastric acid output, chemotherapy with 5-fluoroaracil and streptozotocin, and surgical excision of all resectable tumours (however, the success rate seems very low). In fact, gastrinomas of MEN1 syndrome are frequently multiple and usually include a malignant component; in about 50% of patients gastrinomas have already metastasised before the diagnosis and 30% of patients die [23]. Patients with liver metastases have a poor prognosis for survival. Prognosis does not seem negatively influenced by nodal metastases. Pancreatic gastrinomas are more aggressive than duodenal gastrinomas due to their larger size and greater risk for hepatic metastases.

#### Insulinomas

These insulin-secreting tumours arise in about 10% of MEN1 patients, often in association with gastrinomas, and have approximately one decade earlier onset than that of sporadic insulinomas [16]. Pancreatic insulinoma is characterised by fasting hypoglycaemia; biochemical analysis reveals increased plasma or serum insulin concentration (reference values 2–20 U/ml or 14.35–143.5 pmol/l) together with high plasma or serum concentration of C-peptide (reference values 0.5–2.0 ng/ml or 0.17–0.66 nmol/l) [16]. Since medical control of symptoms is limited, surgery is the main treatment and is curative in some patients. Chemotherapy with streptozotocin or octreotide is used for metastatic disease.

#### VIPoma

These vasoactive intestinal peptide (VIP)-secreting tumours occur as WDHA syndrome, which is characterised by **W**atery **D**iarrhoea, **H**ypokalaemia and **A**chlorhydria. [24]. VIPomas have been reported in a few MEN1 patients only. The diagnosis is made by documenting a markedly increased plasma VIP concentration (reference value <75 pg/ml) [17]. Surgical excision of VIPomas is curative in many cases. In patients with unresectable tumours, medical treatment with streptozotocin, octreotide, corticosteroids, indomethacin, metoclopramide and lithium carbonate has proven beneficial.

### Anterior pituitary tumours

The incidence of pituitary adenomas MEN1 patients varies from 15 to 90%. Generally, symptoms depend on the level of pituitary hormone produced and/or compression effects due to size of the tumour. Mass effects include visual field defects, headaches and blurred vision. Approximately 60% of MEN1-associated pituitary tumours secrete prolactin (prolactinomas), 25% secrete growth hormone (GH) causing gigantism in children and acromegaly in adults, 3% secrete adenocorticotrophin (ACTH) causing hypercortisolism, and the others seem to be non-functional. Pituitary tumours can be detected by computed tomography (CT) scanning and nuclear magnetic resonance imaging (MRI). Treatment of pituitary tumours consists of drug therapy and/or surgery often in association with radiotherapy of the residual unresectable tumours.

#### Prolactinoma

These prolactin-secreting tumours are the most common pituitary tumours in MEN1 and they are characterised by the increased serum concentrations of prolactin (reference values: premenopausal women 0–20 ng/ml; postmenopausal women 0–15 ng/ml; men 0–15 ng/ml) [17]. Prolactinomas induce galactorrhoea, amenorrhoea and infertility in women, and hypogonadism, sexual dysfunction and, more rarely, gynecomastia in men. Medical treatment consists of dopamine agonists such as cabergoline, bromocriptine, pergolide and quinagolide [25].

### Associated endocrine tumours

#### Adrenal cortical tumours

The incidence of adrenal cortical tumours (involving one or both adrenal glands) in MEN1 patients has been reported to be approximately 20–40% [2]. The majority of these tumours are non-functioning. However, functioning adrenal cortical tumours are associated with elevated serum concentrations of cortisol causing hypercortisolaemia and Cushing's syndrome. Although general agreement does not exist, some authors recommend surgical removal of adrenocortical tumours greater than 3 cm in diameter because of their malignant potential.

#### Thyroid tumours

Thyroid tumours, consisting of adenoma, colloid goitres and carcinomas have been reported to occur in over 25% of MEN1 patients [13]. As the prevalence of thyroid disorders in the general population is high, the occurrence of thyroid lesions in MEN1 patients may be incidental and not significant.

### Non-endocrine associated tumours

#### Carcinoid tumours

These tumours are estimated to occur in about 10% of MEN1 patients and may be located in the bronchi, the gastrointestinal tract, the pancreas or the thymus. Thymic carcinoids are more prevalent in males than in females [26], while bronchial carcinoids are more prevalent in females than in males. Carcinoids are generally silent and most patients are asymptomatic. Rarely, thymic, bronchial and gastric carcinoids oversecrete ACTH, calcitonin, GHRH, serotonine or histamine, and rarely cause carcinoid syndrome. Carcinoids can be detected and localised by X-ray CT examination.

#### Facial angiofibromas

Multiple facial angiofibromas have been observed in 88% of MEN1 patients [6]. They are benign tumours comprising blood vessels and connective tissue, and consist of acneiform papules that do not regress.

#### Facial collagenomas

These multiple, skin-coloured, sometimes hypopigmented cutaneous nodules have been reported in >70% of MEN1 patients [6]. They manifest in a symmetrical arrangement on the trunk, neck and upper limbs. They are typically asymptomatic, round-shaped and firm-elastic in nature, and can range from few millimetres to several centimetres in size.

#### Lipomas

Lipomas occur in 20–30% of MEN1 patients [6]. They are generally multiple benign fatty tissue tumours that are subcutaneous or, rarely, visceral. When surgically removed, they usually do not recur.

Cutaneous tumours (angiofibromas, collagenomas, lipomas) may help the presymptomatic diagnosis of MEN1, before manifestations of hormone-secreting tumours appear.

#### Meningiomas

They are mainly asymptomatic and in 60% of cases show no growth.

## Molecular genetics of MEN1 syndrome

### The *MEN1 *gene

The gene locus causing MEN1 has been localised to chromosome 11q13 by studies of loss of heterozigosity (LOH) on MEN1-associated tumours and by linkage analysis in MEN1 families [27-30]. The results of these studies agree with Knudson's "two hits" model for tumour development [31] and indicated that the *MEN1 *gene is a putative tumour suppressor gene. The mutated *MEN1 *allele is a germline mutation present in all cells at birth. The second mutation is a somatic mutation that occurs in the predisposed endocrine cell and leads to loss of the remaining wild type allele; it gives cells the survival advantage needed for tumour development.

In 1997 the responsible gene, *MEN1*, was identified by positional cloning [32]. It spans about 10 Kb and consists of ten exons encoding a 610 amino acid nuclear protein, named menin. Mutation analysis revealed that the *MEN1 *gene was frequently, but not always, mutated in MEN1 families [32]. To date, more than 400 different germline or somatic mutations have been reported in MEN1 families and sporadic cases from several international studies. Mutations are distributed over the entire coding region without showing any significant hot spot region [33-38]. Approximately 20% of mutations are nonsense mutations, about 50% are frameshift insertions and deletions, 20% are missense mutations and about 7% are splice site defects. The nonsense mutations and many of the frameshift insertions and deletions and donor-splice site mutations are truncating mutations predicting a loss-of-function of menin, and therefore supporting the hypothesis that *MEN1 *is a tumour suppressor gene. About 68% of identified missense mutations occur on an amino acid that is conserved among humans, mice, zebrafish and *Drosophila*. More than 10% of the *MEN1 *mutations arise *de novo *and may be transmitted to subsequent generations. Nevertheless, about 10–20% of MEN1 patients may not harbour mutations within the coding region of the *MEN1 *gene [15,33-35,39]; these individuals may have mutations in the promoter or untranslated regions (UTRs), which remain to be investigated. Moreover, because all MEN1 families investigated to date have tight linkage to 11q13, the presence of another tumour suppressor gene in this region is also a possibility [40].

### The MEN1 protein (menin)

*MEN1 *gene encodes a 610 amino acid (67 Kda) nuclear protein that is highly conserved among humans, mice (98%) and rats (97%), and more distantly among zebrafish (75%) and *Drosophila *(47%) [41-45]. Analysis of the menin amino acid sequence did not reveal homology to any other known protein, sequence motif or signal peptide, thus the putative function of menin could not be deduced. Since the amino acid sequence and mutation profile of menin provide a few clues to the functions of menin, most of what is known about its role is derived from *in vitro *studies. These studies revealed that menin is located primarily in the nucleus [46] and identified at least two independent nuclear localisation signals (NLSs) in the C-terminus of the protein. None of the *MEN1 *missense mutations or in-frame deletions [3,15,33-36,47-50] alter either of these NLSs. However, all truncating mutations induce a lack of at least one of these NLSs. The nuclear localisation of menin suggests that this protein may have an important role in the regulation of DNA transcription and replication, in cell cycle, or in the maintenance of genome integrity. Recent studies have demonstrated that over-expression of menin in a Ras-transformed NIH3T3 cell model reversed the transformed phenotype [51], inducing decreased proliferation, suppression of growth in soft agar and inhibition of tumour growth in nude mice. There is increasing evidence that menin may act in DNA repair or synthesis, but the exact mechanism by which menin regulates DNA synthesis or DNA repair in response to DNA damage, is currently unknown. In the last years menin has been shown to interact with several proteins of known functions.

The first identified partner of menin was JunD, a transcriptional factor belonging to the AP1 transcription complex family. Menin interacts with the N-terminus of JunD through its N-terminus and central domains (which are critical for this interaction). Wild type menin represses JunD-activated transcription maybe *via *a histone deacetylase-dependent mechanism [52,53].

Menin interacts, directly, with three members of the nuclear factor NF-**k**B family of transcription regulators: NF-**k**B1 (p50), NF-**k**B2 (p52) and RelA (p65) [54]. These proteins modulate the expression of various genes and are involved in the oncogenesis of numerous organs. Menin interacts with NF-**k**B by its central domain and represses NF-**k**B-mediated transcription.

Moreover, menin interferes with the Transforming Growth Factor beta (TGFβ) signalling pathway at the level of Smad3. Alteration of the TGFβ signalling pathways is important in pancreatic carcinogenesis.

Even the rodent protein Pem has been shown to bind menin directly [55]. Pem is a homeobox-containing protein which plays a role in the regulation of transcription. However, since Pem sequence has no known homolog in the human genome, its direct relevance to MEN1 in humans is still controversial. Mouse and human menin are very similar and this could suggest the existence of a human protein, with a function similar to that of Pem, which binds menin and thus plays a role in the pathogenicity of *MEN1 *mutations.

Although menin has been identified primarily as a nuclear protein, recent studies have reported its interaction with the glial fibrillary acid protein (GFAP) and with vimentin (components of intermediate filaments (IFs)), suggesting a putative role in glial cell oncogenesis.

Finally, menin interacts with the metastasis suppressor Nm23H1 [56]. This interaction enables menin to act as an atypical GTPase and to hydrolyze GTP. The binding of menin to Nm23H1 may be relevant also to the control of genomic stability, as Nm23H1 is associated to the centrosome that is involved in the maintenance of chromosome integrity. This may be supported by the fact that normal cells from MEN1 patients present an elevated level of chromosome alterations [57-60] and that MEN1 tumours have more genome aberrations than equivalent tumours from non-MEN1 patients [61].

## Management

As many of the organs at highest risk of tumour development in MEN1 syndrome such as duodenum, pancreas and lungs (bronchial carcinoids) are not suitable for preventive ablative surgery, routine surveillance of asymptomatic MEN1 at-risk individuals by biochemical analysis and imaging procedures (beginning in early childhood and continuing for life) is recommended. In fact, early detection and treatment of the potential malignant neuroendocrine tumours should reduce the morbidity and mortality of MEN1 syndrome. Such screenings can detect the onset of the disease about ten years before symptoms develop and thus provide an opportunity for earlier treatment.

According to the International Guidelines for Diagnosis and Therapy of the MENs syndromes [16] the minimal surveillance program for individuals known to have MEN1 syndrome or to have a family-specific mutation of the *MEN1 *gene should include:

1) biochemical evaluation of serum concentration of prolactin from age 5;

2) biochemical screening of fasting total serum calcium concentration (corrected for albumin) from age 8;

3) biochemical screening of fasting serum gastrin concentration from age 20;

4) magnetic resonance imaging (MRI) of the head from age 5 and every 3–5 years;

5) abdominal CT or MRI from age 20 and every 3–5 years;

It should also be considered:

1) biochemical screening of fasting serum concentration of full-length PTH;

2) yearly chest CT;

3) yearly somatostatin receptor scintigraphy (SRS)

4) yearly Octreotide scan.

Individuals who have a 50% risk of having MEN1 syndrome, but whose genetic status is unknown, should undergo the following tests:

1) biochemical evaluation of serum concentration of prolactin from age 5;

2) biochemical screening of fasting total serum calcium concentration (corrected for albumin) from age 10;

3) biochemical screening of fasting serum gastrin concentration, if the individual has symptoms of ZES (reflux, diarrhoea), from age 20;

4) biochemical screening of fasting serum concentration of full-length PTH from age 10.

In addition, it is possible to perform prophylactic thymectomy to prevent thymic carcinoids [16]; it should be considered at the time of neck surgery for PHPT, particularly in men with MEN1 syndrome who are smokers and/or have relatives with thymic carcinoids [62].

## Genetic diagnosis/genetic counselling

MEN1 is a monogenic syndrome and, according to its dominant pattern of inheritance, each affected patient has the 50% of probability of transmitting the gene defect to the progeny, independently by sex. Although uncommon, this syndrome is important to be early recognised because the gene mutations confer a high risk of multiple primary tumours occurring at younger ages. Because of the growing number of preventive care options available to MEN1 patients and families, the early clinical and genetic identification of at-risk individuals is becoming increasingly important.

Early recognition of affected and at-risk individuals is now facilitated by DNA-testing [16], reducing the morbidity and mortality of MEN1 and providing the opportunity to initiate treatment at early stages. In fact, since genetic screening was introduced in 1997, the identification of MEN1 in at-risk individuals has become possible even in the absence of more than one affected gland, or before the manifestation of MEN1 typical lesions in first degree relatives. Mutational analysis of the *MEN1 *gene is recommended for patients who meet the clinical criteria for MEN1 and for those in whom a diagnosis of MEN1 is suspected. Identification of a mutation in a patient enables testing for relatives. This allows early identification of asymptomatic mutant gene carriers and provides an indication for them to undergo periodic biochemical and/or radiological screening for MEN1-characteristic endocrine tumours. Finding family members without a mutation may lead to a decision for no further screening. Most laboratories currently use direct DNA sequencing strategies of the *MEN1 *gene coding region and intron-exon junctions. This analysis requires a single blood sample, can be performed at any age and does not need, in theory, to be repeated.

When no *MEN1 *gene mutation in a MEN1 pedigree is identified, the genetic confirmation can be achieved by haplotype or linkage analysis of at least two generations of affected members [16]. Haplotype analysis can be performed using specific locus markers flanking the *MEN1 *region and reaches a degree of confidence when a substantial number of affected members have been analysed. Studies on MEN1 families demonstrated private familial haplotype transmission correlated with the disease [63,64].

Nevertheless, the lack of a genotype-phenotype correlation means that neither the localisation nor manifestations of MEN1-associated tumours can be predicted. A wide variability of tumour occurrence and clinical behaviour, even in patients sharing the same *MEN1 *mutation, has been described, making it difficult to foresee the clinical phenotype in asymptomatic mutant gene carriers. MEN1 clinical manifestations, age of onset and natural history, in fact, are widely variable even among members of the same family. The absence of a genotype-phenotype correlation might suggest a possible role of other modifier genes and/or environmental factors. An understanding of the function of *MEN1 *gene and of menin interacting proteins, in the near future, may help correlation studies and assist clinical management of patients.

## Conclusion

MEN1 is a rare Mendelian cancer disease associated with a variety of endocrine and non-endocrine tumours. Although uncommon, early recognition of this syndrome is important because the occurrence of multiple primary tumours at young ages. In 1997 the discovery of the MEN1 causative gene improved the possibility of early identification of affected and at-risk individuals. In the last decade, the increasing knowledge on the molecular and clinical features of MEN1 syndrome, together with the availability of genetic tests, greatly increased the opportunities for intervention and have lead to reduced morbidity and mortality. Further studies on the molecular pathways of the *MEN1 *gene and related protein will help to design novel and more individualised therapeutic modalities, based on genetic information. In fact, although the knowledge of the mechanisms of tumour development in patients with MEN1 has grown tremendously, much work lies ahead. The final goal is to offer patients with *MEN1 *germline mutations an optimal cancer prevention and treatment program.
